# Fast and inexpensive whole-genome sequencing library preparation from intact yeast cells

**DOI:** 10.1093/g3journal/jkaa009

**Published:** 2020-11-27

**Authors:** Sibylle C Vonesch, Shengdi Li, Chelsea Szu Tu, Bianca P Hennig, Nikolay Dobrev, and Lars M Steinmetz

**Affiliations:** 1 Genome Biology Unit, European Molecular Biology Laboratory (EMBL), Heidelberg 69117, Germany; 2 European Molecular Biology Laboratory (EMBL), Protein Expression and Purification Facility, Heidelberg 69117, Germany; 3 Stanford Genome Technology Center, Stanford University School of Medicine, Palo Alto, CA 94304, USA; 4 Department of Genetics, Stanford University School of Medicine, Palo Alto, CA 94304, USA

**Keywords:** yeast, WGS, Tn5, tagmentation

## Abstract

Through the increase in the capacity of sequencing machines massively parallel sequencing of thousands of samples in a single run is now possible. With the improved throughput and resulting drop in the price of sequencing, the cost and time for preparation of sequencing libraries have become the major bottleneck in large-scale experiments. Methods using a hyperactive variant of the Tn5 transposase efficiently generate libraries starting from cDNA or genomic DNA in a few hours and are highly scalable. For genome sequencing, however, the time and effort spent on genomic DNA isolation limit the practicability of sequencing large numbers of samples. Here, we describe a highly scalable method for preparing high-quality whole-genome sequencing libraries directly from *Saccharomyces cerevisiae* cultures in less than 3 h at 34 cents per sample. We skip the rate-limiting step of genomic DNA extraction by directly tagmenting lysed yeast spheroplasts and add a nucleosome release step prior to enrichment PCR to improve the evenness of genomic coverage. Resulting libraries do not show any GC bias and are comparable in quality to libraries processed from genomic DNA with a commercially available Tn5-based kit. We use our protocol to investigate CRISPR/Cas9 on- and off-target edits and reliably detect edited variants and shared polymorphisms between strains. Our protocol enables rapid preparation of unbiased and high-quality, sequencing-ready indexed libraries for hundreds of yeast strains in a single day at a low price. By adjusting individual steps of our workflow, we expect that our protocol can be adapted to other organisms.

## Introduction

Whole-genome sequencing is a powerful tool in genomics research by providing an unbiased and comprehensive view of the genetic alterations present in a cell. Genomic information is instrumental for identifying mutations that underlie observed phenotypes and for pinpointing any collateral damage that can occur as a side product of mutagenesis. As sequencing costs continue to drop, the cost and time for preparation of sequencing libraries have become the major limiting factor for large-scale genome sequencing experiments.

The most rapid and scalable library preparation methods use a hyperactive variant of the Tn5 transposase that fragments double-stranded DNA and ligates synthetic oligonucleotide adapters required for Illumina sequencing in a 5-min reaction (Adey *et al.* 2010) (Illumina). While the one-step tagmentation reaction greatly simplifies library preparation workflows compared to traditional, multistep methods, and scales to the parallel processing of hundreds of samples, the cost of commercial reagents prevents its use in large-scale projects for most laboratories. We (Hennig *et al.* 2017) and others (Picelli *et al.* 2014) have previously described a robust Tn5 transposase purification strategy and accompanying library preparation protocol that allows generating sequencing libraries with comparable quality but at dramatically reduced cost compared to commercial solutions. In addition to a hyperactive Tn5 enzyme variant carrying the previously reported missense mutations E54K (Zhou and Reznikoff 1997; Zhou *et al.* 1998) and L372P (Weinreich *et al.* 1994), which increase the DNA-binding efficiency and reduce inhibitory effects on Tn5 activity, respectively, we introduced a second Tn5 construct carrying an additional amino acid substitution (R27S) in the DNA-binding domain (Hennig *et al.* 2017), which allows adjusting the fragment size distribution based on enzyme concentration during tagmentation.

With Tn5-based adapter insertion using homemade enzymes, genomic DNA isolation becomes the major bottleneck limiting the practicability of sequencing large numbers of genomes. Analogous to colony PCR, where lysed bacterial cells or yeast spheroplasts are added to a PCR reaction without prior genomic DNA isolation, we hypothesized that yeast whole-genome sequencing library preparation could be simplified by applying tagmentation directly to cells. A similar strategy incorporating heat-based lysis prior to tagmentation was successfully applied for whole-genome (Adey *et al.* 2010) and plasmid (Hwang *et al.* 2019) sequencing of bacterial cells. In contrast to bacteria, the yeast *Saccharomyces cerevisiae* has a rigid cell wall that prevents efficient heat-based lysis. To facilitate cellular lysis the lytic enzyme Zymolyase (Kitamura and Yamamoto 1972), which actively degrades the cell walls of various yeasts, thereby exposing the fragile spheroplast, is routinely used in genomic DNA extraction (van Burik *et al.* 1998; Wilkening *et al.* 2013) and colony PCR (Fuxman Bass *et al.* 2016) protocols. With a molecular weight of 138 kDa the active Tn5 dimer-adapter complex does not far exceed the size of average polymerase enzymes (∼90 kDa), such that treatment with Zymolyase should provide sufficient access to genomic DNA to Tn5. Furthermore, a similar method is used for Tn5-based adapter insertion into accessible DNA in yeast ATAC-seq (Schep *et al.* 2015) protocols.

Here, we report a simplified whole-genome sequencing library preparation protocol. By skipping a conventional genomic DNA isolation step our method enables preparing sequencing-ready libraries directly from overnight yeast cultures in less than 3 h at a cost of 34 cents per sample, while scaling to the parallel processing of hundreds of samples. In place of a lengthy genomic DNA isolation step, we incubate saturated yeast cultures with Zymolyase, followed by heat inactivation to inactivate Zymolyase and lyse spheroplasts, and directly apply tagmentation to the resulting samples. A nucleosome release step prior to enrichment PCR improves the evenness of genomic coverage. Resulting libraries are comparable in quality to libraries processed from extracted genomic DNA with a commercially available Tn5-based kit (Nextera XT, Illumina). We demonstrate the simplicity and unbiased nature of the method by investigating CRISPR/Cas9 on- and off-target editing outcomes in a panel of yeast strains, reliably detecting edited variants, and shared polymorphisms between strains. Direct preparation of sequencing libraries from yeast cultures without DNA isolation enables massively scaled genome sequencing experiments that have so far been hampered by the time and effort spent on genomic DNA isolation. At 34 cents library preparation costs are approximately 5- to 10-fold lower compared to libraries prepared from gDNA extracted using the most simple and scalable kit-based methods. By adjusting steps like initial lysis to specific sample requirements, we anticipate that our protocol can be applied to other microbial cells, mammalian cells, or tissues.

## Materials and methods

### Yeast strain and media

We used the well-characterized yeast strain YJM789 (Wei *et al.* 2007), a derivative of a yeast isolated from the lungs of an AIDS patient with pneumonia, in all experiments. YJM789 contains approximately 60,000 single nucleotide polymorphisms (SNPs) with respect to the *S. cerevisiae* reference genome. For all experiments, we inoculated a YPAD culture directly from glycerol stock and grew it to saturation overnight. For experiments with genomic DNA, we extracted genomic DNA using Master Pure genomic DNA extraction Kit (Epicentre) following the manufacturer’s instructions. We confirmed quality by gel and quantified genomic DNA yield using the Qubit high-sensitivity DNA assay.

### Cell lysis and adjustment of cell numbers

For Zymolyase treatment, cells from overnight cultures were pelleted at 1000 g for 3 min and resuspended in 50 µl of 300 U/ml Zymolyase 100-T (AMSBIO) solution, incubated at 37°C for 30 min followed by 10 min at 95° to inactivate Zymolyase and lyse spheroplasts. About 1.25 µl of the solution was used for tagmentation. Final desired cell number was adjusted prior to Zymolyase treatment by measuring OD_600_ of a diluted overnight culture and calculating sampling volume assuming 30 Mio cells per 1 ml OD 1 culture (based on value from https://bionumbers.hms.harvard.edu/bionumber.aspx?&id=100986&ver=3). Sampling volume was adjusted such that 1.25 µl of the 50 µl Zymolyase reaction contained the desired number of cells.

### Tn5 adapter complex assembly

Tn5 was expressed and purified as previously described (Hennig *et al.* 2017). Tagmentation adapters were annealed as previously described (Hennig *et al.* 2017) and thawed on ice. As Tn5 storage buffer (20 mM Tris pH 7.4, 800 mM NaCl, 50% Glycerol) contains relatively high amounts of salts we mixed 2 µl of Tn5 protein (0.5 mg/ml) with 0.5 µl of each annealed adapter (70 µM stock) and 8 µl of 20 mM Tris pH 7.5 for adapter loading. Providing adapters in slight excess to Tn5 favors all Tn5 dimer molecules to be occupied by two adapters, shifting the equilibrium to the fully saturated Tn5-adapter complex. The mixture was incubated at 23° for 30–60 min at 300 rpm in a thermoshaker. For its use in tagmentation, we further diluted the complex using dilution buffer (10 mM Tris pH 7.5, 150 mM NaCl) to the desired dilution factor.

### Tagmentation with homemade Tn5

Unless indicated otherwise 1.25 µl of lysed cells were mixed with 1.25 µl 1:10 diluted, adapter-loaded Tn5_R27S, E54K, L372P_, and 2.5 µl tagmentation buffer TB1 [10 mM MgCl_2_, 25% dimethylformamide (DMF) (v/v), 10 mM Tris-HCl final concentrations, adjusted to pH 7.6 using acetic acid]. Tagmentation buffer without DMF was prepared as a 2× solution and DMF was added fresh immediately before tagmentation for every experiment. Samples were incubated on a preheated thermocycler block for exactly 3 min at 55°C after which 1.25 µl 0.2% SDS was added immediately for neutralization, and samples were incubated for 5 min at room temperature. For library amplification, we added 1.25 µl each of indexed P5 and P7 primer (10 μM), 6.75 µl of 2× KAPA HiFi Ready mix (KAPA Biosystems), and 0.75 µl of DMSO to 6.25 µl of tagmentation reaction. Salt or Proteinase K (ProK) treated samples were purified using 1.8× volumes of AMPure XP (Beckman Coulter) beads prior to PCR amplification. A gap-filling step followed by 12-cycle enrichment PCR was performed as described previously (Hennig *et al.* 2017) and samples were purified using 1.8× volumes AMPure XP beads and eluted in 10 µl nuclease-free water. We quantified library yield using the Qubit high-sensitivity DNA kit and evaluated library quality on an Agilent Bioanalyzer (high-sensitivity DNA assay).

### Tagmentation with Nextera enzyme

Samples processed from extracted genomic DNA with Nextera XT kit (Illumina) were prepared following kit instructions with the following changes: We used 150 pg DNA as input and scaled down reaction volumes such that we used ¼ of indicated reagents per reaction. For cell-based protocols with Nextera enzyme, tagmentation, inactivation, and library amplification were performed following kit instructions and using commercial reagents, but only using ¼ of indicated reagents. Briefly, 1.25 µl gDNA (100 pg/µl) or zymolyase-treated cells were mixed with 2.5 µl Tagment DNA Buffer (TD) and 1.25 µl Amplicon Tagment Mix (ATM). Samples were incubated on a preheated thermocycler block for exactly 3 min at 55° after which 1.25 µl Neutralize Tagment Buffer (NT) was added and samples were incubated for 5 min at room temperature. Salt or ProK treated samples were purified using 1.8× volumes of AMPure XP (Beckman Coulter) beads prior to PCR amplification. For enrichment PCR, we added 3.75 µl Nextera PCR Master Mix (NPM) and 1.25 µl each of Index N and Index S primers, and ran the NXT PCR program described in the kit instructions. Amplified libraries were purified using 1.8× volumes AMPure XP beads and eluted in 10 µl nuclease-free water. We quantified library yield using the Qubit high-sensitivity DNA kit and evaluated library quality on an Agilent Bioanalyzer (high-sensitivity DNA assay).

### Nucleosome dissociation

We tested two nucleosome dissociation methods. For salt treatment, 6.25 µl of tagmented library was incubated with 3.75 µl of 3 M NaOAc (final 1.125 M) at room temperature for 1 h. For ProK treatment 6.25 µl of tagmented library was incubated with 1.75 µl 0.4 mg/ml ProK (diluted from 20 mg/ml stock) at 65°C for 30 min after tagmentation, unless indicated otherwise. When we applied nucleosome dissociation before tagmentation, we combined cells for several samples using 10 µl of the spheroplast solution as input and eluting in the same volume after bead purification.

### Combined changes

One hundred microliters of a saturated overnight culture (no OD measured) was pelleted and resuspended in 25 µl 300 U/ml Zymolyase solution and incubated at 37° for 30 min, followed by 10 min at 95°. We used 1.25 µl of this solution as input for tagmentation, corresponding to approximately 1.2 Mio cells (assuming 30 Mio cells in 1 ml OD 1 culture and saturation at OD 8). Samples were processed with indicated dilutions of Tn5_E54K, L372P_ or Tn5_R27S, E54K, L372P_ using indicated tagmentation buffers (TB1 or TB2) and tagmentation temperatures (37° or 55°). After tagmentation with the corresponding enzyme and inactivation, samples were incubated with ProK for 30 min at 50° followed by 15 min at 65° and stored at −20° before proceeding with magnetic bead cleanup (1.8×) and enrichment PCR. Final purification was done with 0.8× AMPure XP beads. The final protocol is also described in Supplementary File S1. All samples were pooled and paired-end 150 bp reads were generated on an Illumina NextSeq platform. TB1: 10 mM MgCl_2_, 25% DMF (v/v), 10 mM Tris-HCl final concentrations, adjusted to pH 7.6 using acetic acid. TB2: 8 mM MgCl_2_, 20% DMF (v/v), 16 mM Tris-HCl final concentrations, adjusted to pH 7.6 using acetic acid. Tagmentation buffer without DMF was prepared as a 2× solution and DMF was added fresh immediately before tagmentation.

### Library preparation for on- and off-target editing analysis

Sixteen randomly picked MAGESTIC (Roy *et al.* 2018)-edited yeast strains were inoculated from glycerol stocks into 100 µl YPAD in a 96 well plate, and grown overnight at 800 rpm at 30°. Entire cultures were pelleted at 1000 g for 3 min, pellets were resuspended in 25 µl 300 U/ml Zymolyase solution and incubated at 37° for 30 min, followed by 10 min at 95°. 1.25 µl of the solution was mixed with 2.5 µl TB2 and 1.25 µl of 1:10 diluted Tn5_R27S, E54K, L372P_, and tagmentation was performed at 55°C for 3 min, followed by inactivation with 1.25 µl 0.2% SDS for 5 min at room temperature. For nucleosome dissociation, samples were incubated with 1.75 µl 0.4 mg/ml ProK for 30 min at 50° followed by 15 min at 65° and stored at −20° before proceeding with magnetic bead cleanup (1.8×) and enrichment PCR. Final purification was done with 0.8× AMPure XP beads. All samples were pooled and paired-end 150 bp reads were generated on an Illumina NextSeq platform.

### Whole-genome sequence analysis

Each whole-genome sequencing dataset was down-sampled to consistent numbers of reads for further comparisons, using bash command “gunzip -c *reads.fastq.gz* | head -n *N.*” For comparisons that involved both samples with 150 and 75 bp reads all reads were trimmed to length 75 prior to downstream processing. 3ʹ Transposon adapter sequences were trimmed off using cutadapt (Martin 2011) and trimmed paired-end reads mapped to the S288c yeast reference genome (sacCer3 R64.2.1) using *bwa* (Li and Durbin 2009) (v0.7.17). The bam files containing all mapped reads were sorted and PCR duplicates filtered using an embedded version of *picard* toolkits in *gatk* v4.0 (McKenna *et al.* 2010). Base quality score recalibration (BQSR) was performed using “gatk BaseRecalibrator” to detect and correct systematic errors in the original base accuracy scores. Statistics for assessing mapping qualities were generated using samtools (Li *et al.* 2009) (v1.9) with its “flagstat” option. Insert size distribution was extracted using the command “gatk CollectInsertSizeMetrics.” Variant calling was performed using “gatk HaplotypeCaller” with the haploid option “-ploidy 1.” Low-quality variant calls were annotated and filtered using “gatk VariantFiltration” with expression “QD < 2.0 ‖ FS > 60.0 ‖ MQ < 40.0 ‖ SOR > 3.0 ‖ MQRankSum < −12.5‖ ReadPosRankSum < −8.0”.

### SNP-calling rate assessment

We used the set of variants called in a sample processed with the commercial Nextera pipeline as gold standard (number of total true SNPs), and determined true and false-positive SNP-calling rates with each protocol variation. For any given sample *X*, the true positive rate was defined as (number of correctly called SNPs/number of total true SNPs), while the false-positive rate was defined as (number of miscalled SNPs/number of total SNPs in *X*).

### Nucleosome occupancy and GC content bias analysis

We used two datasets of yeast nucleosome mapping (Lee *et al.* 2007; Schep *et al.* 2015). For tiling-array data, nucleosome occupancy was assessed by the log ratio of probe signals between mono-nucleosomal DNA and total genomic DNA samples. For ATAC-seq data, we calculated transposon insertion frequency per base using *pyatac* (Schep *et al.* 2015) (integrated in *nucleoatac*).

We grouped base positions of the rDNA locus into four categories: 35S rRNA genes, external transcribed spacers, internal transcribed spacers, and nontranscribed regions (NTS), according to published gene annotations of the S288c reference genome (http://sgd-archive.yeastgenome.org/sequence/S288C_reference/genome_releases/S288C_reference_genome_R64-2-1_20150113.tgz).

GC content was calculated for the yeast autosomal genome with a 500 bp sliding window stepped by 250 bp. We took the mean value from overlapped windows to represent GC content bias for single-base position. For correlation tests (coverage *vs* nucleosome occupancy, coverage *vs* GC content), we randomly subsampled 100,000 positions to reduce computational complexity.

### Off-target variant analysis

For the analysis of 16 strains edited by MAGESTIC, read mapping and variant calling were performed as described above, except: (1) gvcf files were generated using “gatk HaplotypeCaller -ERC GVCF” before genotyping in cohort mode; and (2) additional filters were applied to exclude variants with low read depth (<0.1 × *sample average base coverage*) and variants with missing genotypes in ≥2 samples. Mutant allele frequency (MAF) was calculated by counting fraction of nonreference alleles after excluding missing genotypes. For each private mutation (unique variants present in single strain), we scanned for gRNA-like sequences in both strand directions of a (60+*X*) bp (*X* equals variant length) window centered at variant position. We measured the Levenshtein edit distance between on-target sequence and its best match in the (60+*X*) bp window.

Data of *in vitro* on- and off-target sequence pairs were downloaded from the CIRCLE-seq publication (Tsai *et al.* 2017) and edit distance between each pair was calculated accordingly.

### Data availability

The pETM11-Sumo3-Tn5 plasmids carrying either the Tn5_E54K, L372P_ or Tn5_R27S, E54K, L372P_ construct are available upon request. Supplementary File S1 contains detailed step-by-step instructions of the protocol using homemade Tn5-based and nucleosome dissociation by salt or ProK. Supplementary File S2 contains Supplementary Figures S1–S8. Supplementary Table S1 contains protocol conditions and associated insert sizes. Supplementary Table S2 contains editing outcomes for edited strains. Supplementary Table S3 contains genotype information for edited strains. The sequencing data discussed in this manuscript have been deposited on SRA and are accessible through the accession number SRP282188. 

Supplementary material is available at figshare DOI: https://doi.org/10.25387/g3.13187504.

## Results

### Initial comparison of libraries prepared from genomic DNA *vs* intact cells

**Figure 1:**
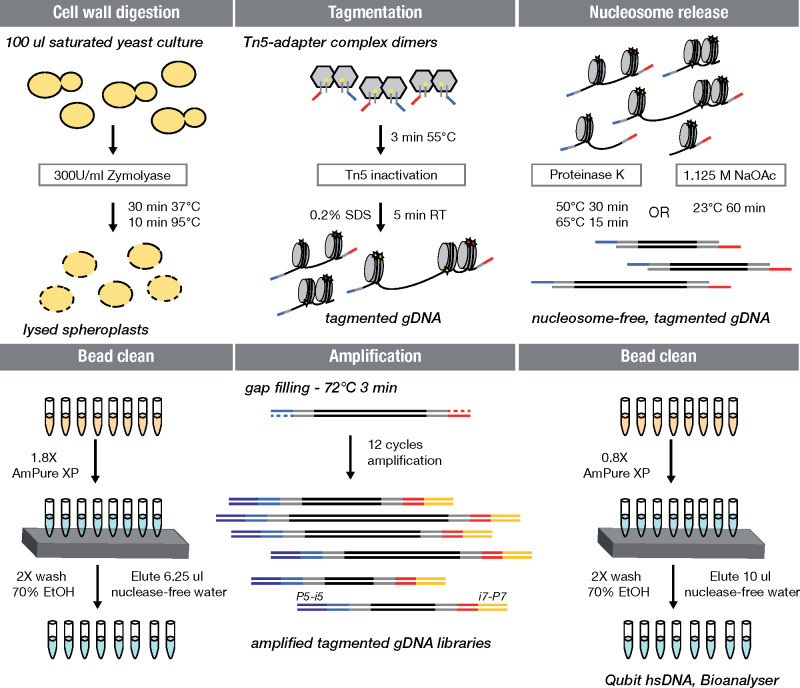
A simplified whole-genome sequencing library preparation workflow without genomic DNA isolation. Workflow of cell wall digestion and heat-based lysis, gDNA tagmentation, nucleosome dissociation, and subsequent NGS library preparation for dual index (i5/i7) whole-genome sequencing is depicted. The double-stranded part of the linker oligonucleotide is shown in gray with a yellow dot depicting the phosphorylated 3′ end. The 5′ overhangs serving as templates for the indexed P5 (dark blue) or P7 (orange) adapter primers are shown in blue and red, respectively.

We hypothesized that tagmentation could be applied directly to heat-treated yeast spheroplasts to simplify whole-genome sequencing library preparation by skipping genomic DNA isolation. Figure 1 illustrates the extraction-free library preparation workflow using homemade Tn5_R27S, E54K, L372P_. Instead of extracting genomic DNA, cells from saturated overnight cultures are exposed to Zymolyase treatment to digest the cell wall, incubated at 95° for inactivation of Zymolyase and lysis of spheroplasts, and the Tn5 adapter complex is added directly to the sample (Figure 1). As nucleosomes could provide a barrier for polymerase during library amplification, we compared two methods for nucleosome dissociation: salt or ProK treatment. High salt concentrations promote dissociation by decreasing the attractive force between positively charged histone proteins and negatively charged DNA (Stacks and Schumaker 1979; Yager *et al.* 1989) while ProK is a broad-spectrum serine protease (Bajorath *et al.* 1988) used for crosslink release and nucleosome digestion in MNase assays (Yuan *et al.* 2005). We performed all experiments with the well-characterized yeast strain YJM789 containing approximately 60,000 SNPs relative to the yeast reference genome (Wei *et al.* 2007). In an initial attempt, we used 240,000 cells corresponding to approximately 3 ng genomic DNA as input for tagmentation. For comparison, we performed extraction-free preparation with the commercial Nextera enzyme, using buffers and reagents from the kit (Nextera XT, Illumina). As a reference standard, we prepared a library from 150 pg extracted YJM789 genomic DNA using reagents from the kit (NXTMP). Paired-end 75-bp reads were generated on an Illumina MiSeq platform and reads mapped to the reference yeast genome (sacCer3 R64.2.1) using *bwa* (Li and Durbin 2009).

A major concern when skipping genomic DNA isolation is the reduced accessibility of genomic DNA to Tn5 and polymerase, leading to a greater variation in coverage across the genome (coverage bias). To assess the impact on the distribution of genomic coverage, we quantified the number of reads mapping to each position, as well as average genome coverage for each sample. We used the ratio of per-base coverage to average coverage to illustrate coverage bias—the closer this ratio is to 1, the more evenly the base is covered relative to the rest of the genome. As a secondary bias metric, we calculated the fraction of the genome covered at least eightfold, reflecting a common variant calling filter. To measure the impact of coverage variation on variant calling, we determined the fraction of true positive SNPs and the false-positive call rate with increasing sequencing coverage using the extracted sample (NXTMP) as a reference.

**Figure 2:**
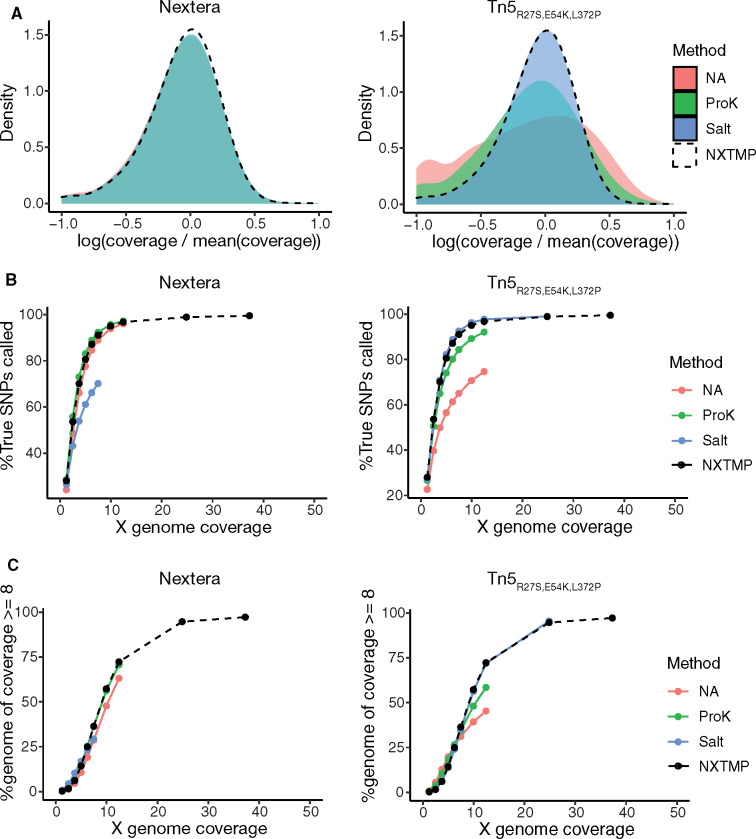
Comparison of different nucleosome dissociation methods in extraction-free library preparation. Samples starting from 240,000 cells were processed following the workflow depicted in Figure 1. After tagmentation nucleosomes were dissociated using salt (blue) or ProK (green). NA (salmon) indicates the lack of a nucleosome dissociation step. NXTMP (black dashed line) is a library processed from 150 pg extracted genomic DNA with the Nextera XT kit and serves as reference standard. (A) Coverage bias distribution (log scale), with bias calculated as coverage at a base divided by average genome coverage. The salt condition is omitted for Nextera due to insufficient reads for this sample. (B) Fraction of YJM789 SNPs called as a function of sequencing depth. The reference set of true positive SNPs (52,373 SNPs) is derived from variant calling on a sample prepared from extracted genomic DNA with the Nextera XT kit (NXTMP). (C) Fraction of the genome covered at least 8× as a function of sequencing depth.

With the Nextera enzyme, libraries prepared from cells without nucleosome dissociation (NA) were highly similar to libraries prepared from extracted genomic DNA (Figure 2, Supplementary Figure S1), and a ProK nucleosome dissociation step after tagmentation did not provide an additional benefit. Despite equimolar pooling, we obtained very low read numbers for the sample with salt treatment and could not evaluate it across the entire range, but at lower sequencing coverage salt treatment seemed to negatively affect variant calling performance (Figure 2B). With homemade Tn5_R27S, E54K, L372P_ in contrast, both nucleosome dissociation methods improved coverage (Figure 2, A and C, Supplementary Figure S1A) and SNP-calling rate (Figure 2B, Supplementary Figure S1B) compared to no treatment (NA) when applied after tagmentation. Salt treatment resulted in libraries that were indistinguishable in quality from the extracted sample. Insert size distribution (median/mode) was similar for the extracted sample (NXTMP 115/70) and extraction-free samples with salt treatment, while ProK treatment resulted in larger insert sizes for both Nextera (150/96) and Tn5_R27S, E54K, L372P_ (182/139) (Supplementary Figure S1A, Table S1).

The native Tn5 transposase has an integration site preference (Goryshin *et al.* 1998; Shevchenko 2002; Reznikoff 2008) and studies have reported a mild GC bias *in vitro* (Green *et al.* 2012) and in library preparation applications (Lan *et al.* 2015). To assess sequence-dependent bias in coverage, we calculated nucleotide composition of the yeast reference genome in 500-bp sliding windows. No correlation with GC content was apparent for either the extracted sample or our extraction-free method using the Nextera kit (Supplementary Figure S1C). The absence of PCR-dependent GC bias, as reported previously (Adey *et al.* 2010), could be a result of using the GC tolerant KAPA HiFi polymerase (KAPA Biosystems). Salt-treated libraries prepared with the extraction-free protocol and homemade Tn5_R27S, E54K, L372P_ showed a mild GC bias (Supplementary Figure S1C). Overall, these initial experiments showed that high-quality whole-genome sequencing libraries can be generated without a genomic DNA isolation step with both commercial and homemade transposase enzymes.

### Optimizing protocol parameters with homemade Tn5

Tagmentation tends to generate libraries with shorter insert sizes than methods using mechanical or enzymatic fragmentation (Adey *et al.* 2010), which can be a limitation for applications requiring longer inserts. Compared to Tn5-based standard library preparation from genomic DNA, library preparation from cells with ProK-mediated nucleosome dissociation consistently produced longer fragments, providing an opportunity to address this need. To identify robust conditions for an efficient, extraction-free workflow with our in-house transposase compatible with longer insert sizes, we extensively varied different reaction steps. To assess whether providing Tn5 with a nucleosome-free DNA substrate would improve genome coverage, we applied ProK treatment after Zymolyase incubation but prior to tagmentation. Compared with treatment after tagmentation, we observed larger variation in genome coverage and lower performance for other quality metrics (Supplementary Figure S2) for both Nextera and Tn5_R27S, E54K, L372P_. The overall worse performance could stem from a higher loss of material during bead-based purification after ProK treatment, as intact genomic DNA might not bind and elute as efficiently as smaller, tagmented genomic DNA, but we did not investigate this further. With 65° at the higher end of the permissible temperature range for ProK activity, we tested incubation at 50°, and 50° followed by 15 min at 65°. Both variations improved coverage bias (Supplementary Figure S3A) and to a smaller degree variant calling (Supplementary Figure S3, B and C) compared to incubation at 65°, but the 50° treatment by itself led to reduced insert sizes (Supplementary Figure S3D, Table S1) and reduced genomic coverage at lower sequencing depths (Supplementary Figure S3E). A combined incubation at 50° followed by 65° resulted in highest library quality while retaining larger insert sizes.

**Figure 3:**
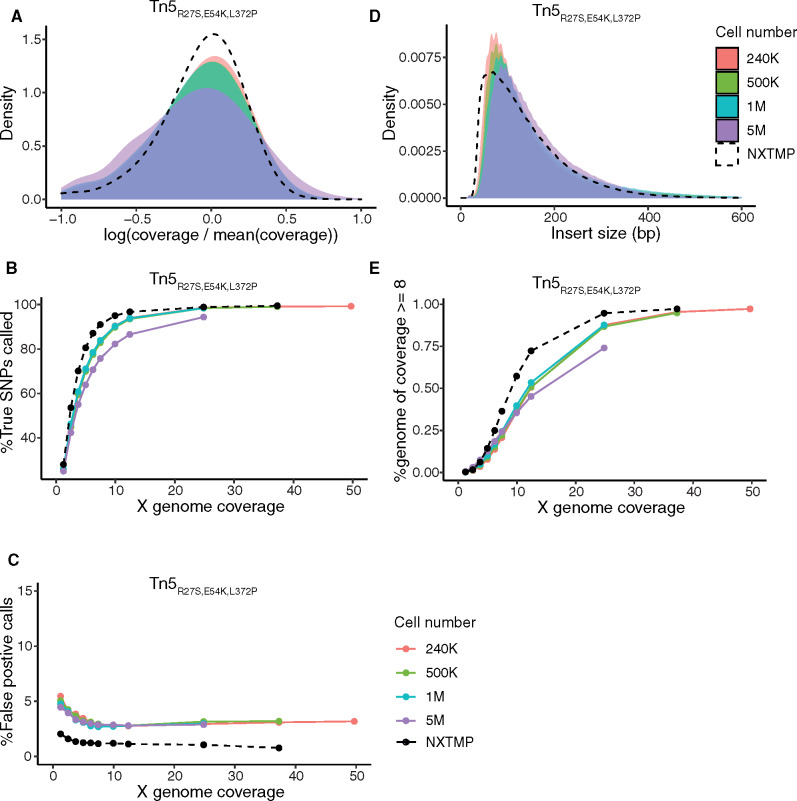
Extraction-free library preparation is robust to variation in input cell number. Samples starting from 240,000 (salmon), 500,000 (green), 1 million (turquoise), or 5 million (purple) cells were processed using homemade Tn5_R27S, E54K, L372P_ and nucleosome dissociation by incubation with ProK after tagmentation. Cell numbers between 240,000 and 1 million yield stable results in terms of library quality. (A) Coverage bias distribution (log scale), with bias calculated as coverage at a base divided by average genome coverage. (B) Fraction of YJM789 SNPs called as a function of sequencing depth. The reference set of true positive SNPs (52,373 SNPs) is derived from variant calling on a sample prepared from extracted genomic DNA with the Nextera XT kit (NXTMP). (C) Fraction of false-positive SNP calls as a function of sequencing depth, with false-positive call rate = number of miscalled SNPs/total number of SNPs. (D) Distribution of fragment insert sizes. Final cleanup used 1.8× Ampure beads. (E) Fraction of the genome covered at least 8× as a function of sequencing depth. NXTMP (black dashed line) is a library processed from 150 pg extracted genomic DNA with the Nextera XT kit and serves as reference.

For initial testing, we measured optical density and adjusted cell number of individual cultures, which limits throughput for parallel processing. To evaluate the robustness of the protocol to variable input cell numbers, we processed samples starting from 240,000 to 5 million cells. We adjusted sampling volume from the saturated culture such that 1.25 µl of the Zymolyase reaction contained the desired number of cells, to prevent any changes in the volumes of reactions. Libraries generated from 240,000, 500,000, and 1 million cells (corresponding to approximately 3, 6, and 12 ng of input DNA) were highly similar in terms of coverage bias (Figure 3, A and E), variant calling (Figure 3, C and D), and insert size distributions (Figure 3D). The main effect of increasing cell number was an increased library yield after enrichment PCR. Increasing input cell number to 5 million cells (∼60-ng DNA), in contrast, increased coverage bias and resulted in lower SNP-calling rate, potentially due to an unfavorable ratio of Tn5 to DNA molecules or insufficient lysis at higher cell densities. The robustness of the protocol toward cell number variation facilitates parallel processing of many samples as it makes it unnecessary to measure optical density and adjust cell number of individual samples.

Previous studies have reported that magnesium chloride and DMF concentrations in the tagmentation buffer affect performance (Picelli *et al.* 2014). As our tagmentation buffer (TB1: 10 mM MgCl_2_, 25% DMF and 10 mM Tris-HCl, pH 7.6) was optimized for cDNA libraries, we varied buffer composition to improve tagmentation of genomic DNA from lysed spheroplasts. We used 500,000 cells in these tests and omitted the nucleosome dissociation step to evaluate the effect of buffer changes only. Lowering the DMF concentration to 20% led to a slight reduction in coverage bias (Supplementary Figure S4, A and E) and improved variant calling performance (Supplementary Figure S4, B and C) to almost the same level as the extracted sample. Lowering DMF further to 17.5%, 15%, or 10% (only 10% shown in plot) had no clear benefit compared to 20% DMF. In addition to DMF, we altered the concentration of both magnesium chloride and Tris-HCl for an optimized tagmentation buffer TB2 containing 8-mM MgCl_2_, 20% DMF, and 16-mM Tris-HCl, pH 7.6 (final concentrations).

**Figure 4:**
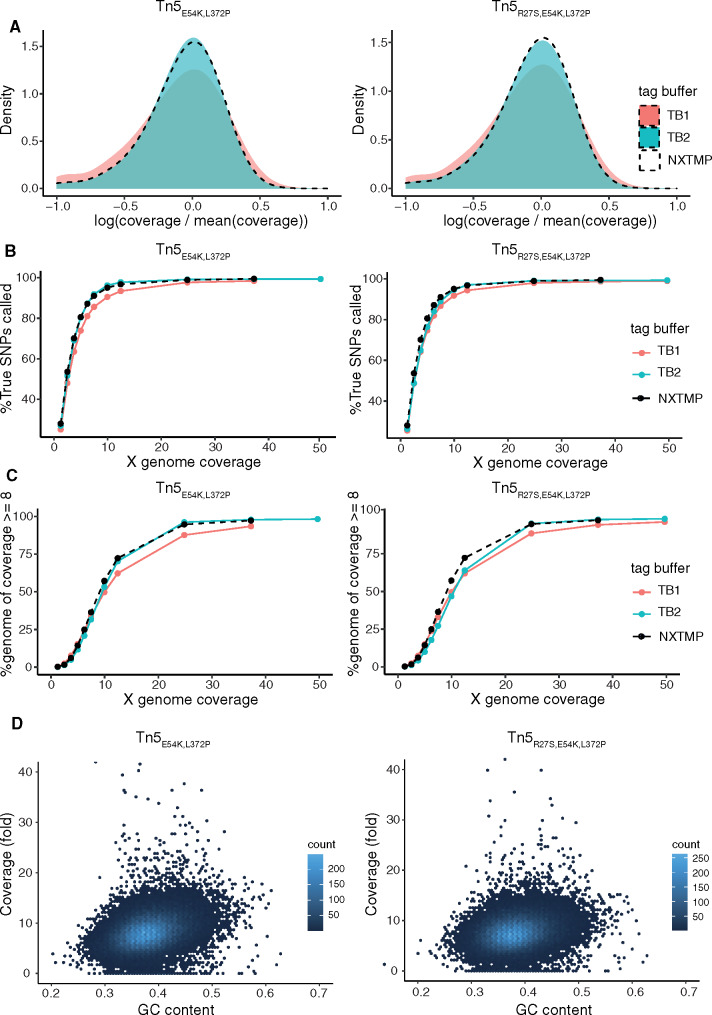
An extraction-free protocol using homemade enzymes produces libraries of the same quality as a commercial, extraction-based solution. Samples were prepared from 100 µl saturated overnight culture with nucleosome dissociation by ProK treatment at 50° followed by 65° and tagmentation buffer TB1 (salmon) or TB2 (turquoise), and with homemade Tn5_E54K, L372P_ or Tn5_R27S, E54K, L372P_ enzyme. (A) Coverage bias distribution (log scale), with bias calculated as coverage at a base divided by average genome coverage. (B) Fraction of YJM789 SNPs called as a function of sequencing depth. The reference set of true positive SNPs (52,373 SNPs) is derived from variant calling on a sample prepared from extracted genomic DNA with the Nextera XT kit (NXTMP). (C) Fraction of the genome covered at least 8× as a function of sequencing depth. NXTMP (black dashed line) is a library processed from 150 pg extracted genomic DNA with the Nextera XT kit and serves as reference standard. (D) GC content related coverage bias extraction-free libraries. GC content of the *S. cerevisiae* reference genome was determined for 500-bp sliding windows with 250 bp overlap, and per-base GC content was calculated as the average of overlapping windows.

As both altered ProK and tagmentation conditions led to small but discernible improvements in library quality individually, we next evaluated them in combination. Taking advantage of the robustness of the protocol to variations in input cell number, we resuspended pellets of 100 µl saturated overnight cultures directly in 25 µl 300 U/ml Zymolyase solution, without measuring the optical density of the culture, and used 1.25 µl of this solution, corresponding to approximately 1.2 Mio cells, as input for tagmentation. After tagmentation and inactivation, we incubated each sample with ProK for 30 min at 50° followed by 15 min at 65°, and stored samples at −20° before proceeding with magnetic bead cleanup and enrichment PCR. As our two different homemade Tn5 enzyme versions (Tn5_E54K, L372P_ and Tn5_R27S, E54K, L372P_) had previously shown different characteristics (Hennig *et al.* 2017), we compared their performance to address whether one would be superior to the other. Compared with tagmentation in buffer TB1, coverage and variant calling were clearly improved with buffer TB2 for both transposase versions, resulting in libraries indistinguishable in quality parameters from the sample prepared from extracted genomic DNA (Figure 4, A–C, Supplementary Figure S5B) while retaining longer insert sizes (Supplementary Figure S5A, Table S1). No correlation with GC content was apparent in extraction-free libraries prepared with either Tn5_R27S, E54K, L372P_ or Tn5_E54K, L372P_ (Figure 4D). To assess whether we could further modulate the insert size distribution specifically toward longer inserts, we evaluated alternative tagmentation parameters. We had previously found that higher enzyme dilutions, which decrease the ratio of Tn5 to DNA molecules, resulted in a progressive shift of the insert size distribution toward larger inserts for libraries made from cDNA (Hennig *et al.* 2017). We observed a similar effect for libraries prepared with our extraction-free protocol. Using a fivefold higher enzyme dilution, we could shift insert sizes from 158/112 (median/mode) to 211/186 (Supplementary Figure S6, Table S1). Tn5 transposase is typically used at 55° in library preparation applications. Lowering tagmentation temperature to 37° resulted in larger insert sizes (192/149), indicating reduced activity at this temperature, without affecting library quality (Supplementary Figure S6). In summary, we identified optimized nucleosome dissociation and tagmentation conditions, as well as strategies to generate libraries with larger insert sizes than is common for Tn5-based adapter insertion. We also demonstrated that our protocol is robust to variation in input cell numbers, which facilitates parallel processing of many samples.

### Detailed characterization of libraries prepared from genomic DNA *vs* intact cells

We extensively compared the genomic characteristics of libraries generated with the extraction-free protocol, using Tn5_R27S, E54K, L372P_ processed libraries with TB2 as an example, with standard library preparation from extracted genomic DNA (NXTMP). Coverage of individual positions across the genome was relatively well correlated between standard and extraction-free methods. Positions with high coverage in standard extraction-based library preparation (NXTMP) tended to have high coverage in extraction-free samples, with the notable exception of a population dropping in coverage in the sample without genomic DNA extraction (Supplementary Figure S7A). We observed the same trend for extraction-free preparation using Nextera (with ProK treatment, from Figure 2), indicating the coverage loss was specific to extraction-free protocols and not related to the use of different Tn5 enzymes. We mapped these positions to specific sites in the yeast rDNA locus, which occurs in 100–200 tandem-arrayed 9.1 kb repeats on chromosome XII (Nomura *et al.* n.d.; Sandmeier *et al.* 2002), making up almost 10% of the genome. The rDNA locus exists in two distinct chromatin states (Merz *et al.* 2008), an accessible and a highly condensed state, and only a small fraction of the repeats is expressed in normal growth conditions, which could explain the reduced coverage for these regions. This is consistent with a higher variability in coverage across the 37S rRNA gene of the locus specifically in extraction-free samples (Supplementary Figure S7B). Across the whole genome, we observed no correlation between coverage and nucleosome positions, as evaluated by ATAC-seq insertion frequency (Schep *et al.* 2015) (Supplementary Figure S7C) or nucleosome occupancy (Lee *et al.* 2007) (Supplementary Figure S7D), and standard and extraction-free samples again looked very similar. A few outlier positions with distinctly higher coverage in the extraction-free sample, specifically in regions with a nucleosome occupancy score of zero (Supplementary Figure S7D), also mapped to the rDNA locus, which shows a bias toward higher coverage of NTS in extraction-free samples (Supplementary Figure S7B). Taken together, we conclude that our extraction-free protocol using homemade Tn5 enzymes generates whole-genome sequencing libraries that are similar in quality to libraries prepared from extracted genomic DNA using a commercial kit, at significantly reduced cost and improved throughput and with the additional benefit of generating longer inserts.

### CRISPR-Cas9 on- and off-target activity profiling in yeast

**Figure 5:**
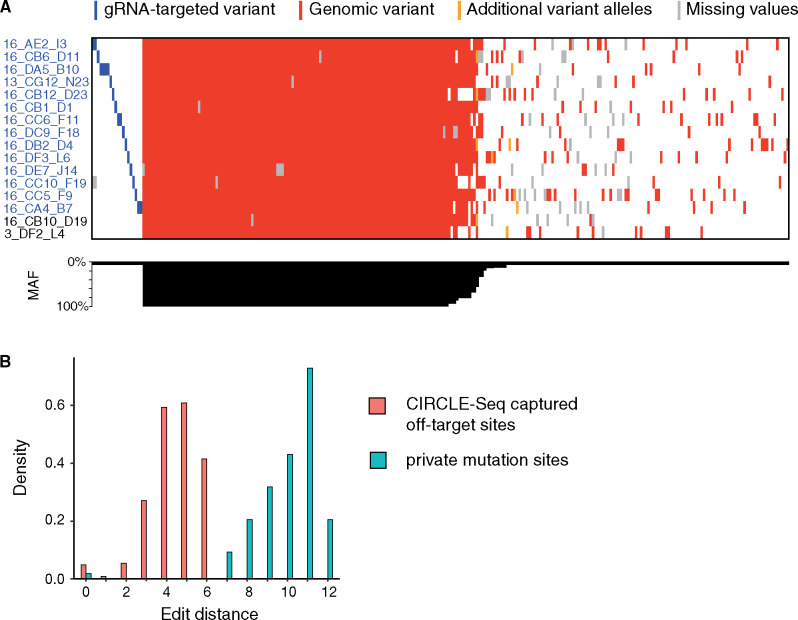
On- and off-target editing analysis in extraction-free libraries. (A) Overview of identified mutations in each edited strain (indicated on *y*-axis), at a sequencing depth of 20×. Designed target variants are shaded in blue. Additional genomic variants are indicated in red or orange, with orange sites representing alternative, non-wild-type genotype calls at sites at which an alternative allele has already been detected, likely representing false-positive variant calls. Sites at which no genotype information could be obtained (no coverage) are highlighted in gray. The *x*-axis depicts a histogram of MAF of each variant. (B) Histogram of edit distances between sequence windows surrounding gRNA targeted variants and alternative variants, reflecting sequence similarity between sites. Sequence similarity between experimentally captured CIRCLE-seq off-target variants (salmon) and their respective gRNA targeted variant indicate expected edit distances if a variant is caused by Cas9-mediated off-target activity. Edit distances obtained for private mutations in each strain in our dataset are depicted in turquoise.

We applied our method to validate the presence of designed variants and identify any unwanted off-target mutations in a set of 16 yeast strains edited with MAGESTIC, our pooled CRISPR guide-donor method for massively parallel precision editing and genomic barcoding (Roy *et al.* 2018). Unique DNA barcodes present at the genomic barcode locus tag a designed variant in each strain (Supplementary Table S2). We constructed sequencing libraries of these 16 strains and performed 150PE sequencing, generating an average of ∼20-fold coverage per genome. After read mapping, variant calling, and variant filtering, we detected 131 SNPs and 145 small insertions or deletions across all samples (Figure 5A). Of the 16 strains, 14 received the desired mutations while 2 carried the wild-type allele at the targeted locus. Among the 276 called variants, 121 were background variants (MAF = 1) representing the baseline genetic differences between the S288c-derivative editing base strain and the S288c reference genome, while 107 were private variants, uniquely present in single lineages (Supplementary Table S3).

Unintended mutations caused by Cas9 off-target activity should be private mutations, given the uniqueness of each guide RNA sequence and the dependence on sequence similarity to the target (Doench *et al.* 2016). To assess the likelihood of each variant to be derived from an off-target cleavage event, we quantified the sequence similarity of the window surrounding each private mutation to the relevant target sequence (20 nt gRNA + 3 nt PAM). As a positive control set, we calculated the edit distances between known on- and off-target sequence pairs captured by CIRCLE-Seq (Tsai *et al.* 2017) and compared them to the spectrum of edit distances in our dataset. There was a clear difference between the two datasets, with experimentally captured off-target sites from CIRCLE-seq exhibiting edit distances of 0–6 to the target site, while all but one of our private variants showed edit distances of seven or higher (Figure 5B). Higher edit distances in the CIRCLE-Seq data, corresponding to more mismatches in the off-target relative to the on-target site, were associated with lower cleavage activity (Supplementary Figure S8). This further supports that the observed private mutations were unlikely to be caused by Cas9 off-target activity, but rather derived from spontaneous mutation events, accumulated in the cell divisions since editing. In summary, we used our protocol to generate whole-genome sequencing libraries directly from saturated cultures of CRISPR-edited yeast strains, and detected designed on-target, background, and spontaneous mutations in all strains. The fact that the majority of the identified mutations were either common (MAF = 1) or private, even at a moderate sequencing depth of 20×, indicates that our extraction-free method generated high-quality, unbiased whole-genome sequencing libraries with even coverage across the genome while substantially simplifying the library construction process and reducing cost.

## Discussion

We describe a simplified whole-genome sequencing library preparation workflow to generate high-quality, sequencing-ready libraries directly from yeast cultures without genomic DNA isolation. Enzymatic digestion of the yeast cell wall followed by brief heat treatment of spheroplasts is sufficient for Tn5 to access genomic DNA, and removal of residual nucleosomes prior to enrichment PCR can improve the evenness of genomic coverage, such that resulting libraries are indistinguishable in quality from libraries prepared from extracted genomic DNA with a commercial kit (Nextera XT). With our method, the time from saturated yeast culture to library is reduced to less than 3 h, enabling massively scaled sequencing projects by eliminating the time- and labor-intensive genomic DNA isolation step while preserving library quality. The robustness of the protocol toward input cell number variation further facilitates parallel processing of many samples as it makes it unnecessary to measure optical density and adjust sampling volumes of individual cultures. In addition to its simplicity and the benefits in throughput, the protocol is highly affordable with reagent costs of 34 cents per sample when using our homemade Tn5 enzymes (Hennig *et al.* 2017). This is an approximately 5- to 10-fold reduction compared to samples prepared from gDNA extracted using the most simple and scalable kit-based methods. Combining our method with low-coverage (3×) sequencing allows genotyping of segregant panels or determining targeted genome mutagenesis outcomes for only $1 per genome. We believe that these advances will enable massively scaled genome sequencing experiments that have so far been hampered by the time, effort, and cost spent on genomic DNA isolation.

Depending on the individual needs and resources of a lab, we present several options for extraction-free library preparation. If homemade Tn5 is not available, high-quality libraries can be generated using the Nextera XT kit on zymolyase and heat-treated cells, without a nucleosome dissociation step. To save cost, it is sufficient to only use ¼ of indicated reagents. With either of our homemade Tn5 enzymes cost is reduced to 34 cents per sample, and high-quality libraries can be obtained by including a nucleosome dissociation step by salt or ProK. Libraries generated by transposase-mediated adapter insertion (tagmentation) tend to have shorter insert size distributions compared with methods using mechanical or enzymatic fragmentation. Our extraction-free method using ProK-mediated nucleosome dissociation generates longer inserts than extraction-free preparation with no or salt-mediated nucleosome dissociation and than the commercial workflow on genomic DNA, without the need for size selection. We also identify additional tagmentation parameters such as temperature and Tn5 to DNA ratio that allow flexible modulation of insert size for custom applications. Insert size distributions compatible with Illumina systems can be obtained even with relatively high dilutions of the Tn5 enzyme, which further reduces cost of the assay.

With our method, we reliably detected designed on-target and shared background mutations in a panel of edited yeast strains. We could confidently call genotypes at all target sites and detected the majority of mutations, representing differences due to shared genetic background, in all strains. This indicates that our extraction-free method provides high-quality, unbiased genomic information while substantially simplifying the library construction process and reducing cost. While we have only tested this workflow in yeast it should in principle be transferable to other organisms by adjusting initial lysis conditions to the cell type of choice. Adding a homogenization step prior to lysis could further enable direct library preparation from tissues.
